# Comparison of the Biograph Vision and Biograph mCT for quantitative ^90^Y PET/CT imaging for radioembolisation

**DOI:** 10.1186/s40658-020-0283-6

**Published:** 2020-03-04

**Authors:** Britt Kunnen, Casper Beijst, Marnix G. E. H. Lam, Max A. Viergever, Hugo W. A. M. de Jong

**Affiliations:** 1 Department of Radiology and Nuclear Medicine, UMC Utrecht, P.O. Box 85500, GA 3508 Utrecht, the Netherlands; 2 Image Sciences Institute, UMC Utrecht & University Utrecht, Heidelberglaan 100, CX 3584 Utrecht, the Netherlands

**Keywords:** Yttrium-90, PET/CT, Radioembolisation

## Abstract

**Background:**

New digital PET scanners with improved time of flight timing and extended axial field of view such as the Siemens Biograph Vision have come on the market and are expected to replace current generation photomultiplier tube (PMT)-based systems such as the Siemens Biograph mCT. These replacements warrant a direct comparison between the systems, so that a smooth transition in clinical practice and research is guaranteed, especially when quantitative values are used for dosimetry-based treatment guidance. The new generation digital PET scanners offer increased sensitivity. This could particularly benefit ^90^Y imaging, which tends to be very noisy owing to the small positron branching ratio and high random fraction of ^90^Y. This study aims to determine the ideal reconstruction settings for the digital Vision for quantitative ^90^Y imaging and to evaluate the image quality and quantification of the digital Vision in comparison with its predecessor, the PMT-based mCT, for ^90^Y imaging in radioembolisation procedures.

**Methods:**

The NEMA image quality phantom was scanned to determine the ideal reconstruction settings for the Vision. In addition, an anthropomorphic phantom was scanned with both the Vision and the mCT, mimicking a radioembolisation patient with lung, liver, tumour, and extrahepatic deposition inserts. Image quantification of the anthropomorphic phantom was assessed by the lung shunt fraction, the tumour to non-tumour ratio, the parenchymal dose, and the contrast to noise ratio of extrahepatic depositions.

**Results:**

For the Vision, a reconstruction with 3 iterations, 5 subsets, and no post-reconstruction filter is recommended for quantitative ^90^Y imaging, based on the convergence of the recovery coefficient. Comparing both systems showed that the noise level of the Vision is significantly lower than that of the mCT (background variability of 14% for the Vision and 25% for the mCT at 2.5·10^3^ MBq for the 37 mm sphere size). For quantitative ^90^Y measures, such as needed in radioembolisation, both systems perform similarly.

**Conclusions:**

We recommend to reconstruct ^90^Y images acquired on the Vision with 3 iterations, 5 subsets, and no post-reconstruction filter for quantitative imaging. The Vision provides a reduced noise level, but similar quantitative accuracy as compared with its predecessor the mCT.

## Background

New digital positron emission tomography/computed tomography (PET/CT) scanners, such as the GE Discovery MI and the Siemens Biograph Vision, offer better time of flight (TOF) performance and a larger axial field of view (FOV) for higher effective sensitivity than their photomultiplier tube (PMT)-based counterparts. This increased sensitivity can be used to shorten acquisition time, reduce radionuclide activity, and/or improve image quality. It is likely that hospitals will gradually switch to these next generation digital PET/CT scanners.

The higher effective sensitivity of new digital PET/CT scanners could particularly benefit yttrium-90 (^90^Y) imaging, which tends to be very noisy owing to the small positron branching ratio. PET is often regarded as the preferred imaging modality for ^90^Y imaging since it offers higher resolution than Bremsstrahlung single photon emission computed tomography (SPECT) [1, 2]. It is used to image the distribution of ^90^Y microspheres after radioembolisation treatment. Clinical relevant features of a post-treatment ^90^Y PET scan include the lung shunt fraction (LSF), presence of extrahepatic depositions, and intrahepatic dose distribution [2, 3]. At the time of imaging, ^90^Y activities in the liver typically range from 500 to 5000 MBq [4–7]. Since radioembolisation and the dosimetry involved constitute a relatively new clinical area, ^90^Y imaging is often performed in the setting of research studies, where ^90^Y PET is used in dose-response studies [5, 8–10]. Consistency of quantitative measurements is key throughout an imaging study, and a change of scanner during the study may affect this consistency. To warrant study consistency, a good understanding of the ^90^Y imaging properties of both systems is required.

The higher sensitivity of new digital PET/CT scanners may open doors to new applications. Recently, we proposed the use of a low dosage of ^90^Y microspheres (~ 100 MBq) for a pretreatment radioembolisation procedure for therapy planning purposes [11, 12]. Currently, pretreatment radioembolisation procedures are performed with technetium-99m (^99m^Tc)-labelled macro aggregated albumin (MAA). Because of the differences in shape and size between the MAA particles and the microspheres, differences between estimated dose distribution by MAA and the true dose distribution have been reported [4, 13–15]. These differences could be minimized by using the same particle for treatment and pretreatment procedure, as has been shown for holmium-166 (^166^Ho) microsphere radioembolisation [16]. A safe dosage for the pretreatment ^166^Ho microsphere procedure is 250 MBq [17, 18]. Inasmuch as the total energy absorbed per becquerel is higher for ^90^Y than for ^166^Ho, this dosage would translate to 100 MBq of ^90^Y to avoid unintended radiation damage [11, 12, 19].

The low activity of ^90^Y for the pretreatment procedure makes imaging challenging. Using 100 MBq, Bremsstrahlung SPECT can produce quantitative images to accurately estimate the LSF, but images are of low resolution [11]. PET overestimates the LSF at 100 MBq [11]. This is caused by the low count statistics and the high random fraction, which result in a positive bias and high noise levels in the PET reconstruction. This makes PET unsuitable as an imaging modality for imaging the dose distribution of a pretreatment ^90^Y procedure.

The previous study that showed the infeasibility of PET as an imaging modality for a ^90^Y pretreatment procedure was performed with the PMT-based Biograph mCT PET system (Siemens) [11]. Its successor, the digital Biograph Vision, has improved spatial resolution, improved timing resolution, an extended axial FOV, and increased sensitivity by 70% measured at the centre of the transaxial FOV (Table 1) [20]. When comparing fluorine-18 (^18^F) fluorodeoxyglucose images of oncological patients from both systems, the Vision scored higher in terms of overall image quality and image noise, than the mCT [22].

**Table 1:** Technical specifications of Biograph Vision and Biograph mCT

	Vision [20]	mCT [21]
Crystals	LSO, 3.2 × 3.2 × 20 mm	LSO, 4.0 × 4.0 × 20 mm
Detector elements	Silicon photomultipliers	Photomultiplier tubes
Axial FOV	26.1 cm	22.1 cm
TOF timing resolution	210–215 ps	540 ps
Transverse spatial resolution (measured at 1 cm vertically from the centre of the FOV with ^18^F)	3.7 mm (FWHM)	4.4 mm (FWHM)
Sensitivity (according to NEMA NU-2 2012)	16.4 kcps/MBq	10.0 kcps/MBq
Time coincidence window	4.7 ns	4.1 ns
Energy window	435–585 keV	435–650 keV
Bed overlap	49%	43%

*LSO* lutetium oxyorthosilicate, *FOV* field of view, *FWHM* full width at half maximum

The improvements of the Vision with regard to the mCT are expected to lead to improved ^90^Y imaging. The QUEST phantom study [23] has extensively studied the performance of multiple PET systems for ^90^Y imaging and recommends reconstruction settings for quantitative purposes for the systems involved. However, the Vision was not part of the QUEST study and to our knowledge there are no recommended reconstruction settings for this system regarding quantitative ^90^Y imaging.

The purpose of the present study is to evaluate the performance of the Siemens Biograph Vision in comparison with its PMT-based counterpart, the mCT, for ^90^Y imaging. We used the NEMA phantom to determine the ideal reconstruction settings for the Vision in analogy to the QUEST study [23]. These reconstruction settings for the Vision were compared with the recommended reconstruction setting of the mCT [23] in terms of standardized image quality metrics. In addition, clinical relevant features for radioembolisation were compared by using an anthropomorphic phantom.

## Methods

### Phantoms

Two phantoms were used for this study: the NEMA image quality phantom (PTW, Freiburg, Germany) and a modified anthropomorphic thorax phantom (model ECT/TOR/P, IEL, Chilcompton, UK). The NEMA phantom is used to compare standardized image quality metrics in analogy with the QUEST study [23]. The thorax phantom is used to simulate a radioembolisation patient and to evaluate the accuracy of lung shunt estimation, extrahepatic deposition visibility, and intrahepatic activity distribution.

The NEMA phantom consists of a 9.7-L torso-shaped compartment containing six fillable spheres (inner diameter of 10, 13, 17, 22, 28, and 37 mm) and a cold, cylindrical lung insert. It was prepared in a similar way as in the QUEST study protocol [23], where the phantom was filled with ^90^Y chloride, in 0.5 M HCl to prevent adhesion to the plastic phantom walls [24], to acquire a sphere-to-background concentration ratio of 8 and a total initial activity of 2.5·10^3^ MBq.

The modified thorax phantom consists of a torso-shaped compartment containing a liver compartment with a solid tumour (sphere of 15.9 mL) and a necrotic tumour (outer rim 18.9 mL and inner sphere 5.6 mL), two lung compartments, a cylindrical spine insert, and three extrahepatic compartments (spheres of 2.0, 4.2 and 8.2 mL). The phantom was filled with ^90^Y chloride in 0.5 M HCl, to acquire a lung shunt fraction (LSF) of 5.0%, a tumour to non-tumour ratio (T/N) of 8.0, and a total initial activity of 1.0·10^3^ MBq. The activity concentration of the extrahepatic depositions in the phantom was based on the median size (6.8 mL, range 1.1–42 mL) and median activity (1.3% of the infused activity) of 34 extrahepatic depositions found by Prince et al. [18] and was therefore chosen to be 1.3% of the total activity in the phantom/6.8 mL.

### Image acquisition

Both phantoms were scanned consecutively on a Siemens Biograph mCT and a Siemens Biograph Vision. Table 1 lists technical specifications of both systems. Scans were acquired during decay and all activities at the time of imaging are listed in Table 2.

**Table 2:** Total activity (MBq) in the NEMA and thorax phantoms at time of imaging

		Day 0	Day 2	Day 4	Day 6	Day 8	Day 9	Day 10
NEMA	mCT	2511	1560	901	537	328	259	194
	Vision	2563	1526	930	553	322	264	205
Thorax	mCT	961	597	345	206	126	99	74
	Vision	982	585	356	212	123	101	78

Both phantoms were scanned in two bed positions of 15 min per bed position on both systems. A CT scan was made for attenuation correction and to support delineation.

### Image reconstruction

Images acquired by the mCT were reconstructed with the reconstruction settings recommended by the QUEST study [23]. This is an ordered subset expectation maximization (OSEM) reconstruction algorithm, including time of flight (TOF) information, resolution recovery (TrueX), attenuation, scatter, random, dead time, and decay correction. The reconstruction used 2 iterations with 21 subsets, resulting in a voxel size of 4.1 × 4.1 × 3.0 mm^3^, and no post-reconstruction filter was applied.

Since, to our knowledge, no prior study has been published on ^90^Y PET imaging using the Vision, images acquired at the Vision were reconstructed with a variety of reconstruction settings, so as to determine the optimal setting. All reconstruction methods used an OSEM reconstruction algorithm, including TOF information, resolution recovery (TrueX), attenuation, scatter, random, dead time, and decay correction. The number of subsets is fixed at 5 by the vendor and the number of iterations was varied between 1 and 17. Images were reconstructed on a 220 × 220 matrix resulting in a voxel size of 3.3 × 3.3 × 3.0 mm^3^. No post-reconstruction filter was applied. The optimal reconstruction was determined based on convergence of the recovery coefficient with iteration number.

### Analysis

For the NEMA image quality phantom, we analysed the same metrics as the QUEST phantom study. These are:

Percent background variability (BV), following the NEMA NU 2-2007 guidelines, defined for each sphere size as:

1 $$ \mathrm{BV}=\frac{{\mathrm{SD}}_{B,s}}{C_{B,s}}\times 100\% $$

where SD_B,s_ is the standard deviation of the average of the 60 background regions of interest (ROIs) for sphere size *s* and *C*_B,s_ is the average count of the 60 background ROIs for sphere size *s*.

Percentage misplaced counts in the lung insert (∆*C*_lung_), following the NEMA NU 2-2007 guidelines, defined as:

2 $$ \Delta  {C}_{\mathrm{lung}}=\frac{C_{\mathrm{lung}}}{C_B}\times 100\% $$

where *C*_lung_ is the average count in the lung insert ROI and *C*_B_ is the average count of the background ROIs.

Background concentration accuracy (BCA), defined for each sphere size as:

3 $$ \mathrm{BCA}=\frac{a_{\mathrm{B},\mathrm{measured}}-{a}_{\mathrm{B},\mathrm{true}}}{a_{\mathrm{B},\mathrm{true}}}\times 100\% $$

where *a*_B,measured_ is the measured activity concentration in the background and *a*_B,true_ is the true activity concentration in the background.

Total activity accuracy (TAA), defined as:

4 $$ \mathrm{TAA}=\frac{A_{\mathrm{TOT},\mathrm{measured}}-{A}_{\mathrm{TOT},\mathrm{true}}}{A_{\mathrm{TOT},\mathrm{true}}}\times 100\% $$

where *A*_TOT,measured_ is the measured total activity in the entire FOV and *A*_TOT,true_ is the known true total activity in the phantom.

Recovery coefficient (RC), following the NEMA NU 2-2007 guidelines for delineation of the spheres, defined for each sphere size as:

5 $$ \mathrm{RC}=\frac{a_{S,\mathrm{measured}}}{a_{S,\mathrm{true}}}\times 100\% $$

where *a*_S,measured_ is the measured activity concentration in the sphere and *a*_S,true_ is the true activity concentration in the sphere.

To evaluate the thorax phantom, we assessed the following metrics, which are commonly assessed for radioembolisation treatment planning:

The lung shunt fraction (LSF), defined as:

6 $$ \mathrm{LSF}=\frac{C_{\mathrm{lung}}}{C_{\mathrm{lung}}+{C}_{\mathrm{liver}}}\times 100\% $$

where *C*_lung_ is the total count in the lungs dilated by 6 mm and *C*_liver_ is the total count in the liver dilated by 6 mm. The lung and liver contours were dilated by the spatial resolution of the PET systems to partially compensate for the partial volume effect.

Tumour to non-tumour ratio (T/N) of the solid and the necrotic tumour, defined as:

7 $$ \mathrm{T}/\mathrm{N}=\frac{C_{\mathrm{tumour}}}{{\mathrm{C}}_{\mathrm{parenchyma}}} $$

where *C*_tumour_ is the average count in the tumour volume of interest (VOI) and *C*_parenchyma_ is the average count in the parenchymal VOI.

Parenchymal dose per GBq of ^90^Y administered, defined as:

8 $$ {D}_{\mathrm{parenchyma}}=\frac{\frac{A_{\mathrm{parenchyma}}}{A_{\mathrm{total}}}\times 50}{m_{\mathrm{parenchyma}}} $$

where *A*_parenchyma_ is the activity in the parenchymal VOI, *A*_total_ is the known total activity in the phantom, *m*_parenchyma_ is the mass of the parenchymal VOI (determined using the liver VOI volume and a conversion factor of 1.03 g/mL), and 50 is the absorbed energy in joule from the decay of 1 GBq of ^90^Y.

Contrast-to-noise ratio (CNR) of the extrahepatic depositions as a measure of visibility, defined as:

9 $$ \mathrm{CNR}=\frac{C_{\mathrm{deposition}}-{C}_B}{SD_B} $$

where *C*_deposition_ is the average count in the extrahepatic deposition VOI, *C*_B_ is the average count in a background VOI, and SD_B_ is the standard deviation of a background VOI.

Differences between the Vision and the mCT are tested for significance with a paired t-test assuming a 5% significance level.

## Results

**Fig. 1:**
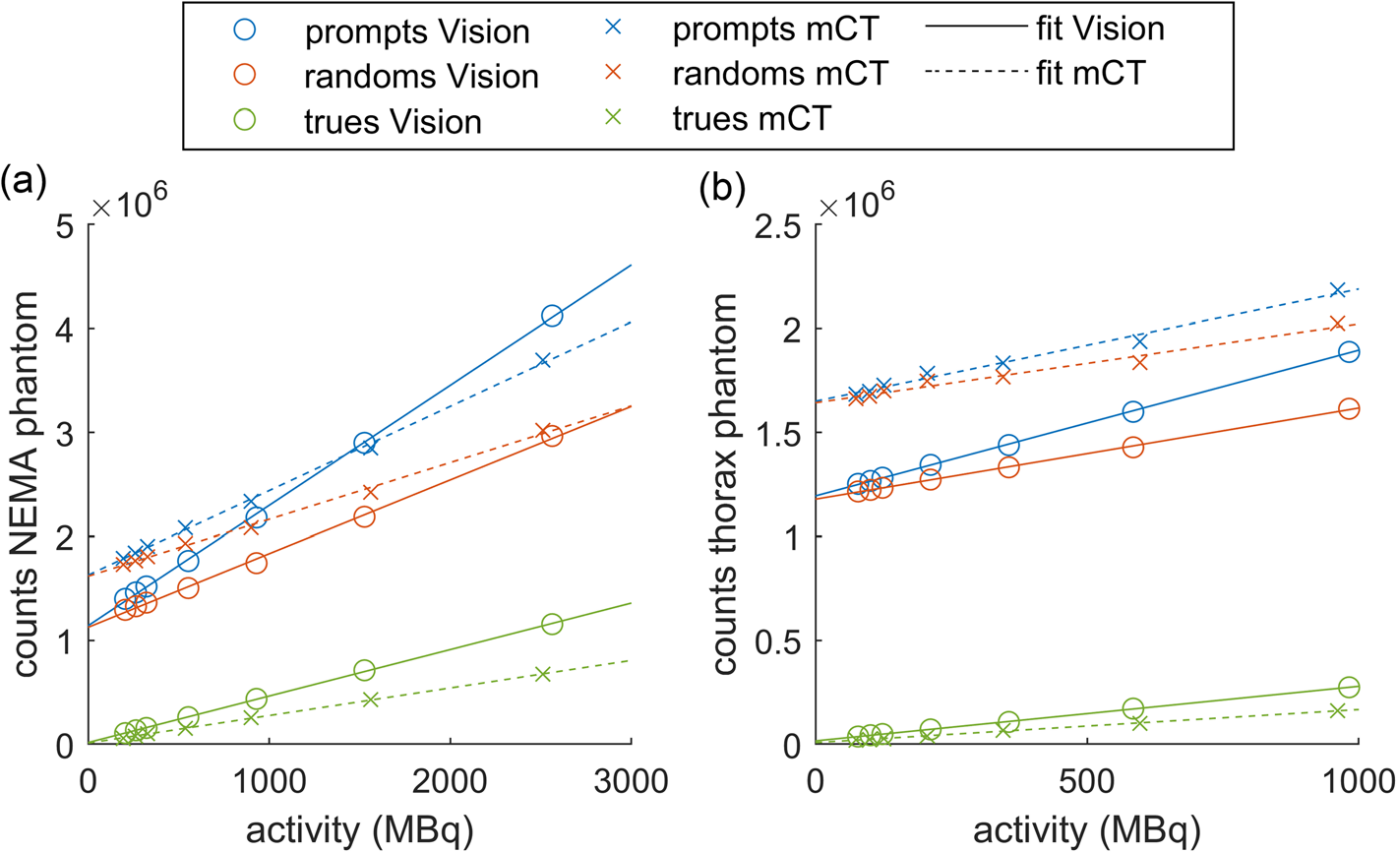
Total prompts, randoms, and net trues for a range of ^90^Y activities for the Vision and mCT acquisitions of the NEMA phantom (**a**) and the thorax phantom (**b**). The solid and dashed lines are linear fits of the data for the Vision and the mCT respectively

Figure 1 shows the total prompts, randoms, and net trues for the Vision and mCT acquisitions of the NEMA and thorax phantoms. The net trues show a strong linear trend with activity, of which the slope, intercept, and coefficient of determination are listed in Table 3. The Vision has an increased trues rate compared with the mCT by 68% (NEMA phantom) and 65% (thorax phantom).

**Table 3:** Slope, intercept, and coefficient of determination (*R*^2^) of the linear fit of the net true counts in the sinograms. Numbers between brackets are the 95% confidence intervals

Measurement	Slope (counts/MBq)	Intercept (counts)	*R*^2^
NEMA Vision	448 [439–457]	1.57·10^4^ [5.16·10^3^–2.623·10^4^]	0.9997
NEMA mCT	266 [261–271]	1.04·10^4^ [4.53·10^3^–1.62·10^4^]	0.9997
Thorax Vision	263 [259–267]	1.58·10^4^ [1.37·10^4^–1.78·10^4^]	0.9998
Thorax mCT	159 [152–167]	0.762·10^4^ [4.15·10^3^–1.11·10^4^]	0.9983

### Ideal reconstruction setting Vision

**Fig. 2:**
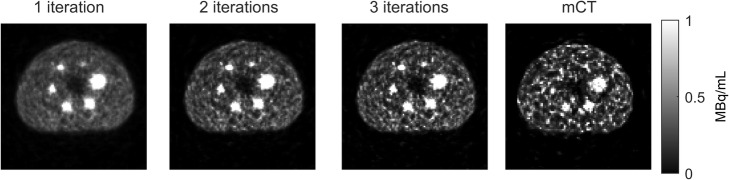
Transverse slice of three Vision reconstructions (1, 2, and 3 iterations with 5 subsets) and mCT reconstruction (2 iterations with 21 subsets) of the NEMA phantom at day 0 (2.5·10^3^ MBq), centred on the spheres. Images are scaled between 0 and 1 MBq/mL

Figure 2 shows the transverse slice of the NEMA phantom at day 0 (2.5·10^3^ MBq) for three reconstruction settings for the Vision acquisition and for the 2-iterations setting for the mCT acquisition. Visually, the reconstructions of the Vision outperform the reconstruction of the mCT because of the lower noise level and the better visibility of the (smaller) spheres. Increasing the number of iterations increases noise.

**Fig. 3:**
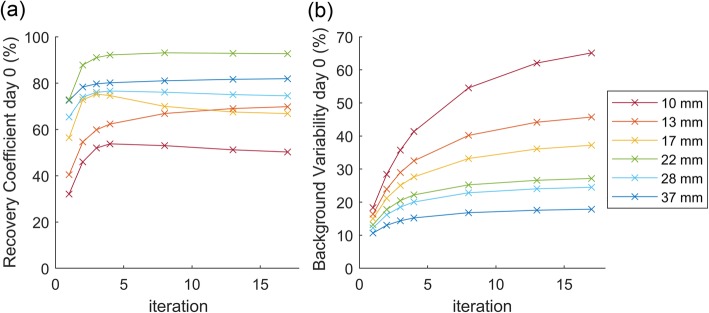
Recovery coefficient (**a**) and percentage background variability (**b**) of the NEMA phantom spheres with varying diameter at day 0 (2.5·10^3^ MBq) as a function of iteration number

Figure 3 shows the recovery coefficient and the percentage background variability plotted against iteration number of the different sphere diameters at day 0. Depending on the study purpose, one can either choose a number of iterations that favours high recovery (higher number of iterations) or one that favours low noise (low number of iterations). Since the purpose of this study is quantitative imaging, we like to achieve a high RC. The RC curves of the three largest spheres shown in Fig. 3 converge at 3 iterations. The RC curves of the three smallest spheres show more varying trends. In order to not increase the background variability while the recovery coefficient barely improves, we choose 3 iterations with 5 subsets as the ideal reconstruction setting for the Vision. *For subsequent comparisons between the Vision and the mCT, the images acquired by the Vision will therefore be reconstructed using 3 iterations with 5 subsets without a post-reconstruction filter*.

### Comparison Vision and mCT

**Fig. 4:**
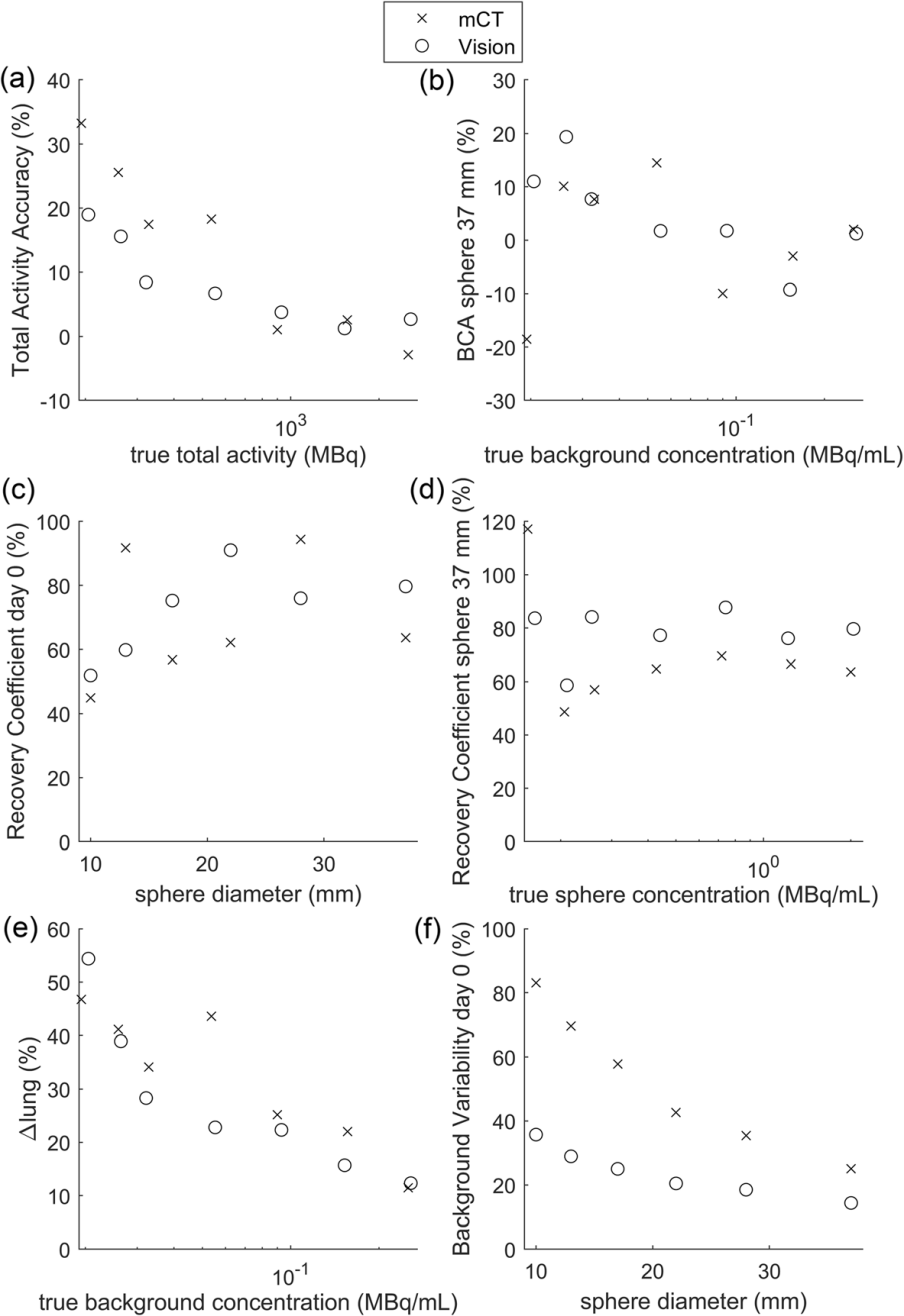
Results of the NEMA phantom comparing the mCT (2 iterations, 21 subsets) with the Vision (3 iterations, 5 subsets). BCA, background concentration accuracy

Figure 4 compares the NEMA phantom scanned at the Vision (reconstructed with 3 iterations, 5 subsets) with the mCT scans (reconstructed with 2 iterations, 21 subsets). Paired *t* tests showed a significant difference between the Vision and the mCT for background variability (BV) (*p* value < 0.001) and background concentration accuracy (*p* value < 0.05). The BV was about twice as high for the mCT. This affects the visibility of hot and cold spots. For other quantitative measures, there was no significant difference between the Vision and the mCT.

**Fig. 5:**
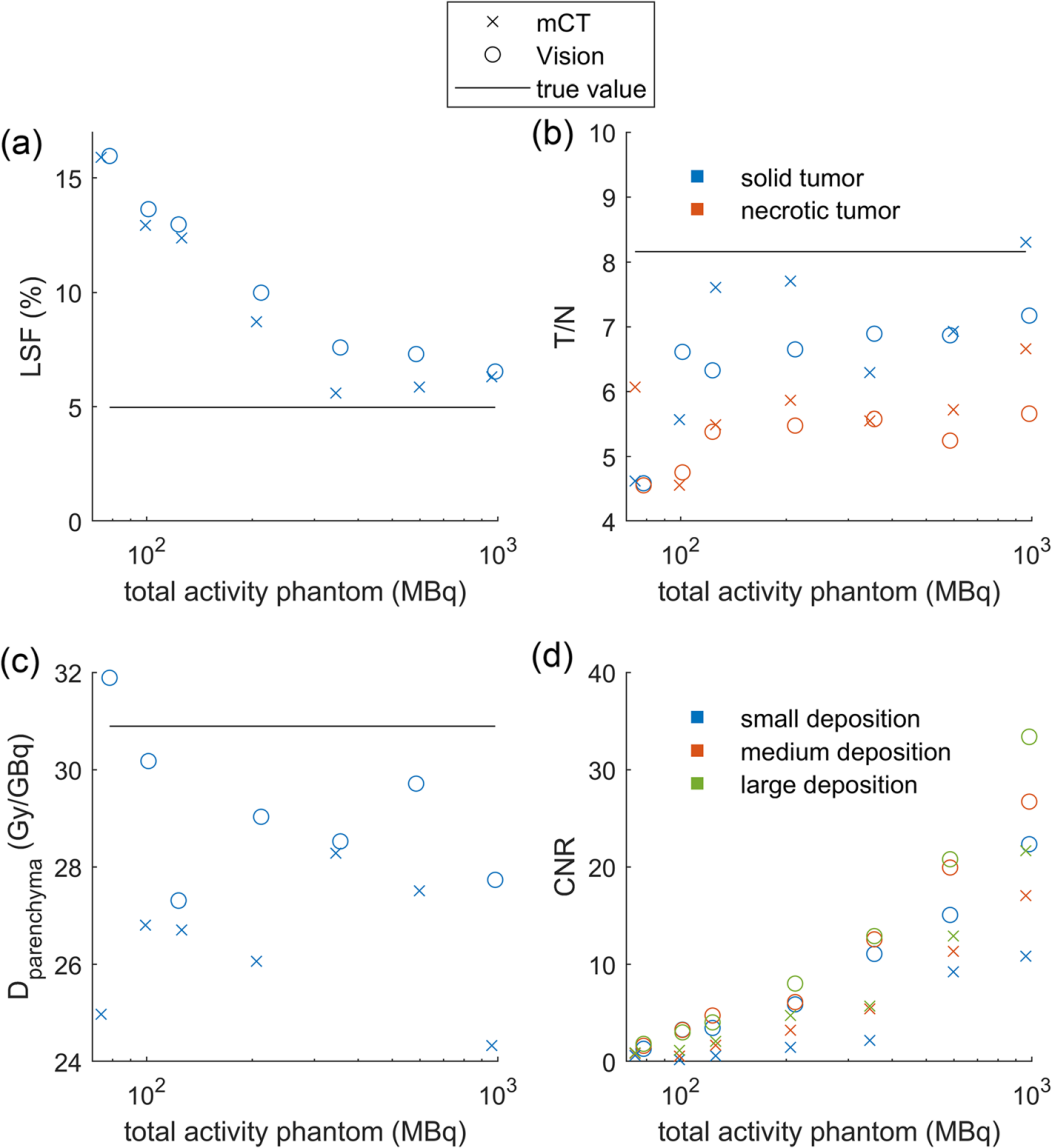
Performance of the Vision and the mCT for measures concerning radioembolisation treatment planning: **a** lung shunt fraction (LSF), **b** tumour to non-tumour ration (T/N), **c** parenchymal dose (*D*_parenchymal_), and **d** contrast-to-noise ratio (CNR) of the extrahepatic depositions

The thorax phantom mimics a radioembolisation patient and has an activity distribution that is clinically more relevant than that of the NEMA phantom. Figure 5 shows metrics that are commonly assessed for radioembolisation treatment planning. Paired *t* tests showed a significant difference between both systems for the LSF (*p* value < 0.05), the parenchymal dose (*p* value < 0.05), and the CNR (*p* value < 0.001). Although significant differences were observed, both systems showed similar trends for these metrics. Both overestimated the LSF, and as expected, the overestimation got worse when the total activity decreased. This can be attributed to the earlier described positive bias in maximum likelihood reconstructions at low activities [11, 25]. However, unexpectedly, the Vision showed a slightly larger overestimation of the LSF than the mCT. Metrics regarding intrahepatic dose distribution (T/N and parenchymal dose) were underestimated by both systems and showed large variations. The CNR, a measure for the visibility of the extrahepatic depositions, showed the most stable trend and decreased with decreasing phantom activity. The CNR was considerably higher for the Vision than for the mCT.

## Discussion

In this study, we evaluated the performance of the Vision for ^90^Y imaging and compared it with the performance of its predecessor, the mCT. We determined the optimal reconstruction settings for the Vision to be 3 iterations with 5 subsets. We found that the Vision outperformed the mCT in terms of noise level, but performed similarly in terms of quantitative accuracy.

The prompts, randoms, and trues of both the Vision and the mCT show good linearity, which implies that no saturation of the detectors occurs. The Vision had 65% (thorax phantom) or 68% (NEMA phantom) more net trues than the mCT. This is in agreement with the 70% increase in sensitivity found by van Sluis et al. for ^18^F [20]. The slope of the true count with activity is dependent on the geometry and activity distribution of the scanned object, since the slopes of the true count with activity on the same scanner with the same acquisition parameters differ between NEMA and thorax phantoms. The total prompts and randoms have an offset due to the presence of lutetium-176 (^176^Lu) in the crystals. The magnitude of this offset is dependent on, e.g. the amount of crystals (and therefore the amount of ^176^Lu), the number of possible coincidence line-of-response, the time coincidence window, and the energy window [26]. All these specifications differ between the mCT and the Vision and result in a larger offset for the mCT than for the Vision.

Scans at both systems were acquired in step-and-shoot mode, instead of continuous-table-motion mode. For scans with a short scan length and long acquisition time, like ^90^Y PET liver scans, it has been shown that step-and-shoot mode has a higher counting efficiency compared to continuous-table-motion mode [27]. Therefore, we chose to perform all scans in step-and-shoot mode to obtain the best image quality and quantification precision.

The ideal reconstruction setting is dependent on the study purpose. Just like the QUEST study, we focused on quantitative accuracy. We recommend to reconstruct ^90^Y data acquired on the Vision with 3 iterations, 5 subsets, and no post-reconstruction filter. This is based on the fact that after three iterations quantitative metrics will only marginally improve while noise will still substantially increase. When the objective of a scan is focused on visual image quality, one could decide to add a post-reconstruction filter. In this study, we did not study the influence of different filters in detail, since Gaussian filters will reduce quantitative accuracy.

The results from our NEMA measurements on the mCT were comparable with the results from the QUEST study. There were some small deviations (like the steep trend seen for our background variability versus sphere diameter, which is less pronounced in the QUEST study), but these can be explained by the fact that the data represented by QUEST consisted of multiple scanners. The measurements on our single scanner still fall within the error margins of the QUEST study.

Comparing quantitative results of the Vision with the mCT does not show clinically relevant differences between the systems. This suggests that a smooth upgrade during quantitative studies is possible, if the proper reconstruction protocol is chosen. However, the noise level of the Vision is substantially lower than the noise lever of the mCT. This could make visualization of hot and/or cold regions, such as extrahepatic depositions, easier on the Vision. van Sluis et al. shows similar results comparing the Vision and the mCT for ^18^F, where they show that the Vision outperforms the mCT visually, but based on quantitative measures, both systems are comparable [22].

For the specific clinical case of ^90^Y imaging for radioembolisation, quantitative measures are important. Although the LSF and parenchymal dose show significant differences between the Vision and the mCT, these differences are small (mean LSF difference is 0.9% point (pp), mean parenchymal dose difference is 2.8 Gy/GBq). Furthermore, the uncertainty in volume measurement and uncertainty in the activity calibration cause an uncertainty of ± 10% in the true parenchymal dose of the thorax phantom. This makes it difficult to conclude with certainty whether the Vision or the mCT results in a more accurate estimated parenchymal dose. With a ^90^Y pretreatment procedure in mind, errors in estimated parenchymal dose could cause under- or overdosage. In combination with the overestimation of the LSF by 9 pp at 100 MBq, we have to conclude that the Vision is, just like its predecessor the mCT, unsuitable as an imaging system for imaging of a theoretically safe pretreatment dosage of 100 MBq ^90^Y.

The Vision does have a lower noise level compared with the mCT. This means that the Vision would be the more appropriate system when addressing the dose distribution visually, for example after radioembolisation treatment to identify whether the dose distribution in a tumour is homogenous or not.

## Conclusion

In this study, we evaluated the performance of the Vision for ^90^Y imaging and compared it with the performance of its predecessor, the mCT. We recommend to reconstruct ^90^Y images acquired on the Vision with 3 iterations, 5 subsets, and no post-reconstruction filter, for quantitative purposes. Visually, the Vision outperforms the mCT because of its lower noise level, but based on quantitative measurements, both scanners perform similarly.

## Data Availability

The datasets used and analysed during the current study are available from the corresponding author on reasonable request.

## Abbreviations

∆*C*_lung_: Percentage misplaced counts in the lung insert
^166^Ho: Holmium-166
^176^Lu: Lutetium-176
^18^F: Fluorine-18
^90^Y: Yttrium-90
^99m^Tc: Technetium-99m
BCA: Background concentration accuracy
BV: Background variability
CNR: Contrast-to-noise ratio
FOV: Field of view
FWHM: Full width at half maximum
LSF: Lung shunt fraction
LSO: Lutetium oxyorthosilicate
MAA: Macro aggregated albumin
OSEM: Ordered subset expectation maximization
PET/CT: Positron emission tomography/computed tomography
PMT: Photomultiplier tube
pp: Percentage point
RC: Recovery coefficient
ROI: Region of interest
SPECT: Single-photon emission computed tomography
T/N: Tumour to non-tumour ratio
TAA: Total activity accuracy
TOF: Time of flight
VOI: Volume of interest

## References

[1] Elschot M, Vermolen BJ, Lam MGEH, de Keizer B, van den Bosch MAAJ, de Jong HWAM (2013). Quantitative comparison of PET and Bremsstrahlung SPECT for imaging the in vivo yttrium-90 microsphere distribution after liver radioembolization. PLoS One.

[2] Pasciak AS, Bourgeois AC, McKinney JM (2014). Radioembolization and the dynamic role of 90Y PET/CT. Front Oncol.

[3] Alsultan AA, Smits MLJ, Barentsz MW, Braat AJAT, Lam MGEH (2019). The value of yttrium-90 PET/CT after hepatic radioembolization: a pictorial essay. Clin Transl Imaging.

[4] Wondergem M, Smits MLJ, Elschot M (2013). 99mTc-macroaggregated albumin poorly predicts the intrahepatic distribution of 90Y resin microspheres in hepatic radioembolization. J Nucl Med.

[5] Dewaraja Yuni K., Devasia Theresa, Kaza Ravi K., Mikell Justin K., Owen Dawn, Roberson Peter L., Schipper Matthew J. (2019). Prediction of Tumor Control in 90Y Radioembolization by Logit Models with PET/CT-Based Dose Metrics. Journal of Nuclear Medicine.

[6] Mazzaferro V, Sposito C, Bhoori S (2013). Yttrium-90 radioembolization for intermediate-advanced hepatocellular carcinoma: a phase 2 study. Hepatology..

[7] Yue J, Mauxion T, Reyes DK (2016). Comparison of quantitative Y-90 SPECT and non-time-of-flight PET imaging in post-therapy radioembolization of liver cancer. Med Phys.

[8] van den Hoven AF, Rosenbaum CENM, Elias SG (2016). Insights into the dose–response relationship of radioembolization with resin 90Y-microspheres: a prospective cohort study in patients with colorectal cancer liver metastases. J Nucl Med.

[9] Kafrouni Marilyne, Allimant Carole, Fourcade Marjolaine, Vauclin Sébastien, Delicque Julien, Ilonca Alina-Diana, Guiu Boris, Manna Federico, Molinari Nicolas, Mariano-Goulart Denis, Ben Bouallègue Fayçal (2018). Retrospective Voxel-Based Dosimetry for Assessing the Ability of the Body-Surface-Area Model to Predict Delivered Dose and Radioembolization Outcome. Journal of Nuclear Medicine.

[10] Lea WB, Tapp KN, Tann M, Hutchins GD, Fletcher JW, Johnson MS (2014). Microsphere localization and dose quantification using positron emission tomography/CT following hepatic intraarterial radioembolization with yttrium-90 in patients with advanced hepatocellular carcinoma. J Vasc Interv Radiol.

[11] Kunnen B, van der Velden S, Bastiaannet R, Lam MGEH, Viergever MA, de Jong HWAM (2018). Radioembolization lung shunt estimation based on a 90 Y pretreatment procedure : a phantom study. Med Phys.

[12] Kunnen B, Dietze MMA, Braat AJAT, Lam MGEH, Viergever MA, de Jong HWAM. Feasibility of imaging 90Y microspheres at diagnostic activity levels for hepatic radioembolization treatment planning. Med Phys. 2020. 10.1002/mp.13974. 10.1002/mp.13974 PMC7078991 31855282

[13] Lambert B, Mertens J, Sturm EJ, Stienaers S, Defreyne L, D’Asseler Y (2010). 99mTc-labelled macroaggregated albumin (MAA) scintigraphy for planning treatment with 90Y microspheres. Eur J Nucl Med Mol Imaging.

[14] Hung JC, Redfern MG, Mahoney DW, Thorson LM, Wiseman GA (2000). Evaluation of macroaggregated albumin particle sizes for use in pulmonary shunt patient studies. J Am Pharm Assoc.

[15] Ilhan H, Goritschan A, Paprottka P (2015). Predictive value of 99mTc-MAA SPECT for 90Y-labeled resin microsphere distribution in radioembolization of primary and secondary hepatic tumors. J Nucl Med.

[16] Smits MLJ, Dassen MG, Prince JF, et al. The superior predictive value of 166Ho-scout compared with 99mTc-macroaggregated albumin prior to 166Ho-microspheres radioembolization in patients with liver metastases. Eur J Nucl Med Mol Imaging. 2019. 10.1007/s00259-019-04460-y. 10.1007/s00259-019-04460-y PMC7075844 31399801

[17] Braat Arthur J. A. T., Prince Jip F., van Rooij Rob, Bruijnen Rutger C. G., van den Bosch Maurice A. A. J., Lam Marnix G. E. H. (2017). Safety analysis of holmium-166 microsphere scout dose imaging during radioembolisation work-up: A cohort study. European Radiology.

[18] Prince JF, van Rooij R, Bol GH, de Jong HW, van den Bosch MA, Lam MG (2015). Safety of a scout dose preceding hepatic radioembolization with 166Ho microspheres. J Nucl Med.

[19] Gulec SA, Mesoloras G, Stabin M (2006). Dosimetric techniques in 90Y-microsphere therapy of liver cancer: the MIRD equations for dose calculations. J Nucl Med.

[20] van Sluis Joyce, de Jong Johan, Schaar Jenny, Noordzij Walter, van Snick Paul, Dierckx Rudi, Borra Ronald, Willemsen Antoon, Boellaard Ronald (2019). Performance Characteristics of the Digital Biograph Vision PET/CT System. Journal of Nuclear Medicine.

[21] Karlberg AM, Sæther O, Eikenes L, Goa PE (2016). Quantitative comparison of PET performance-Siemens Biograph mCT and mMR. EJNMMI Phys.

[22] van Sluis J, Boellaard R, Somasundaram A, et al. Image quality and semi-quantitative measurements of the Siemens Biograph Vision PET/CT: initial experiences and comparison with Siemens Biograph mCT PET/CT. J Nucl Med. 2020;61(1):129–35. 10.2967/jnumed.119.227801 31253742

[23] Willowson KP, Tapner M, Bailey DL, Team Q U E S T investigator (2015). A multicentre comparison of quantitative (90) Y PET/CT for dosimetric purposes after radioembolization with resin microspheres : the QUEST Phantom Study. Eur J Nucl Med Mol Imaging.

[24] Park M-A, Mahmood A, Zimmerman RE, Limpa-Amara N, Makrigiorgos GM, Moore SC (2008). Adsorption of metallic radionuclides on plastic phantom walls. Med Phys.

[25] Van Slambrouck K, Stute S, Comtat C (2015). Bias reduction for low-statistics PET: maximum likelihood reconstruction with a modified Poisson distribution. IEEE Trans Med Imaging.

[26] Conti M, Eriksson L, Rothfuss H (2017). Characterization of176Lu background in LSO-based PET scanners. Phys Med Biol.

[27] Siman W, Kappadath SC (2017). Comparison of step-and-shoot and continuous-bed-motion PET modes of acquisition for limited-view organ scans. J Nucl Med Technol.

